# Laplace Inversion of Low-Resolution NMR Relaxometry Data Using Sparse Representation Methods

**DOI:** 10.1002/cmr.a.21263

**Published:** 2013-05-29

**Authors:** Paula Berman, Ofer Levi, Yisrael Parmet, Michael Saunders, Zeev Wiesman

**Affiliations:** 1 The Phyto-Lipid Biotechnology Laboratory, Departments of Biotechnology and Environmental Engineering, The Institutes for Applied Research, Ben-Gurion University of the NegevBeer-Sheva, Israel; 2 Department of Industrial Engineering and Management, Ben-Gurion University of the NegevBeer-Sheva, Israel; 3 Department of Management Science and Engineering, Stanford UniversityStanford, CA

**Keywords:** low-resolution NMR, sparse reconstruction, *L*_1_ regularization, convex optimization

## Abstract

Low-resolution nuclear magnetic resonance (LR-NMR) relaxometry is a powerful tool that can be harnessed for characterizing constituents in complex materials. Conversion of the relaxation signal into a continuous distribution of relaxation components is an ill-posed inverse Laplace transform problem. The most common numerical method implemented today for dealing with this kind of problem is based on *L*_2_-norm regularization. However, sparse representation methods via *L*_1_ regularization and convex optimization are a relatively new approach for effective analysis and processing of digital images and signals. In this article, a numerical optimization method for analyzing LR-NMR data by including non-negativity constraints and *L*_1_ regularization and by applying a convex optimization solver PDCO, a primal-dual interior method for convex objectives, that allows general linear constraints to be treated as linear operators is presented. The integrated approach includes validation of analyses by simulations, testing repeatability of experiments, and validation of the model and its statistical assumptions. The proposed method provides better resolved and more accurate solutions when compared with those suggested by existing tools. © 2013 Wiley Periodicals, Inc. Concepts Magn Reson Part A 42A: 72–88, 2013.

## I. INTRODUCTION

Low-resolution nuclear magnetic resonance (LR-NMR) relaxometry has emerged as a powerful new tool for identifying molecular species and to study their dynamics even in complex materials. This technology is widely used in industrial quality control for the determination of solid-to-liquid and oil-to-water ratios in materials as diverse as oil-bearing rock, food emulsions, and plant seeds 1. It offers great potential for characterization and ultimately quantification of components in different materials in their whole conformation. Many recent developments are reviewed by Blümich et al. 2. Note that the term LR-NMR is used in other contexts such as time-domain NMR, ex situ NMR, and portable NMR.

The process of relaxation occurs as a consequence of interactions among nuclear spins and between them and their surroundings. In biological samples, spins exist in a variety of different environments, giving rise to a spectrum of relaxation times, where the measured relaxation decay is a sum of contributions from all spins 3. Spin–spin interactions are the main relaxation mechanism in a CPMG (Carr, Purcell, Meiboom and Gill) pulse sequence 4,5.

Conversion of the relaxation signal into a continuous distribution of relaxation components is an ill-posed inverse Laplace transform (ILT) problem. The probability density *f*(*T*_2_) is calculated as follows:


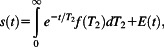
(1)

where *s*(*t*) is the relaxation signal acquired with LR-NMR at time *t*; *T*_2_ denotes the time constants; and *E*(*t*) is the measurements error.

Istratov and Vyvenko 6 reviewed the fundamentals and limitations of the ILT. The most common numerical method implemented today for dealing with ill-posed problems of this kind is based on *L*_2_-norm regularization 3,7–9, where Eq. [1] is approximated by a discretized matrix form and optimized according to the following equation:


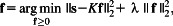
(2)

where *K* is the discrete Laplace transform and λ is the *L*_2_ weight. This type of regularization, however, can significantly distort the solution by contributing to the broadening of peaks.

It should be noted that the non-negativity constraint in Eq. [2] makes the problem much harder to solve. Without the constraint, a standard least-squares (LS) solver can be applied. The solution **f** obtained will satisfy the related normal equation:


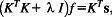
(3)

However, there is no guarantee that **f** will be non-negative even if negative components are not physically feasible, as in the LR-NMR case. In practice, it is not acceptable to set negative values to zero. To solve Eq. [2], optimally, we need more sophisticated optimization tools such as interior-point methods 10.

Sparse representation methods are a relatively new approach for analysis and processing of digital images and signals 11. State-of-the-art optimization tools are used to handle efficiently even highly underdetermined systems. The main feature of these methods lies in using *L*_1_ regularization in addition to the common *L*_2_ regularization. It has been shown in theory and in practice that the *L*_1_ norm is closely related to the sparsity of signals 12. The *L*_1_ norm of the solution is the sum of absolute values of its components. Absolute value terms in the objective function are harder to handle than quadratic terms. However, it is possible to state the *L*_1_-regularized problem as a convex optimization problem and then use an appropriate convex optimization solver. Typically, such solvers can handle the non-negativity constraint.

In this work, we apply advanced sparse representation tools to the problem of LR-NMR relaxometry. We use PDCO, a primal-dual interior method for convex objectives 13. PDCO can be adjusted to solve the LR-NMR relaxometry inverse problem with non-negativity constraints and an *L*_1_ regularization term that stabilizes the solution process without introducing the typical *L*_2_ peak broadening. Our new suggested method makes it possible to resolve close adjacent peaks, whereas existing tools typically fail, as we demonstrate below.

The underlying principle is that all structured signals have sparse representation in an appropriate coordinate system, and using such a system/dictionary typically results in better solutions when the noise level is relatively low. Evidently, one of the most important elements of this approach is choosing an appropriate dictionary.

## II. THE LR-NMR DISCRETE INVERSE PROBLEM

Inverse problems and their solutions are of great importance in many disciplines. Application fields include medical and biological imaging, radar, seismic imaging, nondestructive testing, and more. An inverse problem is typically related to a physical system that can take indirect measurements *s* of some unknown function *f*. The relationship between *s* and *f* is determined by the characteristics of the measurement system and relevant physical principles.

The general setting of an inverse problem in the continuous time domain is as follows:


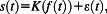
(4)

where *K* is an operator that models the action of the measurement system. The source of the error ε might be machine noise, incorrect or simplified modeling of the system, additional factors or variables that were not included in the model, or varying conditions during different measurements. A schematic description of the system is as follows:


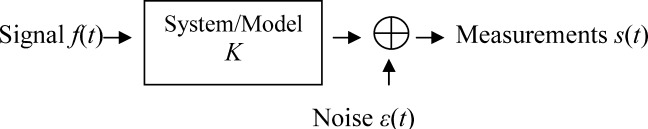


Equation [4] can be used to compute directly the expected measurement function of a known signal *f*. This computation is referred as the forward problem. It does not provide a direct method to estimate the signal *f* given a measurement function *s*. The latter problem is referred to as the inverse problem and requires appropriate optimization tools.

In many cases, as well as for NMR, the relationship between *f* and *s* can be accurately expressed by a linear transformation. For NMR, it is a direct result of the fact that the noiseless model is a Fredholm equation of the first kind—an integral model of the form:


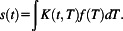
(5)

In this context, *K*(*t*,*T*) is termed as the transformation kernel. One of the main characteristics of such integral transformations is that they are ill posed. An ill-posed problem is one that has one or more of the following properties: a) it does not have a solution; b) the solution is not unique; and c) a small perturbation of the problem may cause a large change in the solution.

Thus, even a low noise might lead to a completely wrong solution.

In practice, the inverse problem at hand is a discrete inverse problem defined as **s** = *K***f** + **e**, where *s* and *e* are *m* vectors and *K* is an *m* × *n* matrix. It is typically advised to choose *n* < *m* and find a LS solution to a tall rectangular system:


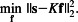
(6)

The exact choice of *n* depends on the nature and conditioning of the matrix *K*. As can be expected, the discrete problem is also ill-posed, and one must be very careful when trying to solve it. Standard methods can lead to very erroneous results because very different functions **f** could correspond to almost the same measurement function **s**.

A common approach is to use regularization methods, which force the solution **f** to possess certain properties. Often one searches for solutions of low magnitude using the *L*_2_ norm; see Eqs. [2] and [3]. This method is known as Tikhonov regularization and typically results in smooth, noise-free solutions. The main drawback is its tendency to oversmooth the solution, and thus inability to detect low-intensity peaks or to resolve between two or more neighboring peaks (which tend to be merged into a single smooth wide peak).

The relationship between the spectrum function *f*(*T*) and the NMR measurements function *s*(*t*) is given by the Laplace transform (Eq. [1]). As can be seen, this is a special case of the Fredholm equation of the first kind (Eq. [5]) with the kernel defined as *K*(*t*,*T*) = exp(−*t*/*T*).

The discrete version of the Laplace transform is defined as *s*_1_ = *s*(*t*_1_), …, *s*_m_ = *s*(*t_m_*), where *t*_1_, …, *t_m_* are the NMR signal acquisition times. The discrete values of **f** are *f*_1_ = *f*(*T*_1_), …, *f_n_* = *f*(*T_n_*), where *T*_1_, …, *T_n_* are the relaxation times, and the elements of *K* are *K_l__,__j_* = exp(−*lΔt*/*jΔT*).

With *m* > *n*, the singular value decomposition (SVD) *K* = *UΣV^T^* solves the LS problem (Eq. [6]) according to the following equation:


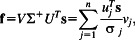
(7)

where *U* and *V* are orthogonal matrices of size *m* and *n*, respectively, and Σ has a lower block of zeros and an upper diagonal block Σ*_n_* = diag(σ_1_, σ_2_, …, σ*_n_*) with the singular values of *K* on its diagonal 14. The singular values are ordered according to σ_1_ ≥ σ_2_ … ≥ σ*_n_* ≥ 0, and the system is ill conditioned when σ_1_/σ*_n_* is large. It can be shown that the error in the solution is as follows:


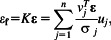
(8)

where **ε** is the vector of measurement errors. Evidently, when *K* has small singular values, small errors in the measurements can result in large errors in the resolved values of **f** because the error is proportional to the reciprocals of the σ*_j_*. Hence, it is a common practice to compress the linear operator *K* by truncating its smallest singular values, making the solution process more stable and less sensitive to measurement errors. This approach was suggested by Song 15 to enable two-dimensional inversions by compressing two one-dimensional inversion matrices before constructing the larger two-dimensional matrix. (Tikhonov regularization is still typically necessary.) The best rank-*r* approximation to *K* is the partial sum of the first *r* SVD components: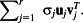 This compression stabilizes the solution while making a relatively small perturbation to the original problem defined by *K*.

Apparently, both *L*_2_ regularization and SVD compression could be applied to improve the condition and stability of the inverse problem as well as to reduce the level of noise in the solution. There is an interesting relationship between the two methods: the *L*_2_ regularization in Eq. [2] is equivalent to applying multiplicative weights to the singular values of *K*, where the weights are given by *w*(σ) = σ^2^/(σ^2^ + λ) 16, and therefore, the larger singular values become more dominant. Thus, *L*_2_ regularization is equivalent to smooth damping of the small singular values, whereas the SVD compression applies sharp truncation to the singular value series.

Other approaches for the NMR spectrum reconstruction include Monte Carlo simulation inversion 17, where an entire family of probable solutions are for a given measurements set. In addition, in Ref. (18, a phase analysis is applied to the measurements function using the Fourier transform to evaluate the exponential decay rates.

Herrholz and Teschke 19 considered sparse approximate solutions to ill-posed inversion problems, using compressed sensing methods, Tikhonov regularization, and possibly infinite-dimensional reconstruction spaces. Their results may be relevant for future work.

## III. THE PROPOSED SOLUTION

The mathematical formulation of our proposed method is the linearly constrained convex optimization problem:


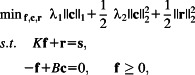
(9)

where *K* is the discrete Laplace transform, **f** is the unknown spectrum vector, ***s*** is the measurements vector, **r** is the residual vector, and *B* is a sparsifying dictionary.

This model is a generalization of the LS model with non-negativity constraints. The objective function includes both *L*_1_ and *L*_2_ penalties on the vector **c**, which is a representation of the solution in a given dictionary *B*. If *B* = *I* (the identity matrix), then **c** = **f** and the sparsity property is imposed on **f** itself. This is most appropriate when the spectrum peaks are expected to be sharp and well localized. The basis pursuit denoising formulation as described in Ref. (11 allows high flexibility in the actual shape of the spectrum peaks. What allows this flexibility is the dictionary *B*. For example, *B* can be chosen to be a wavelet basis and then because of the multiscale property of wavelets, a sparse solution in the wavelet domain can correspond to both thin and thick spectrum peaks, and the optimal solution is expected to represent the actual sample properties. Another efficient choice for *B* might be a dictionary of Gaussians at different locations and with different widths.

Model [9] includes two regularization parameters λ_1_ and λ_2_ as weights on the *L*_1_ and *L*_2_ terms, where λ_1_ controls the solution sparsity in the chosen dictionary *B*, and λ_2_ affects the smoothness of the solution: it can be increased to smooth the solution and to remove noise. In our experiments, ||*K*|| = *O*(1) and ||*B*|| = *O*(1); however, the choice of λ_1_ and λ_2_ must allow for ||***s***|| and ||**r**||. In general, λ_1_ and λ_2_ should be proportional to ||**s**|| and to the level of noise in the measurements: the higher the noise, the larger the regularization parameters.

## IV. METHODS

### The PDCO Solver

PDCO 13,20 is a convex optimization solver implemented in Matlab. It applies a primal-dual interior method to linearly constrained optimization problems with a convex objective function. The problems are assumed to be of the following form:


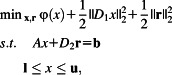
(10)

where **x** and **r** are variables, and *D*_1_ and *D*_2_ are positive-definite diagonal matrices. A feature of PDCO is that *A* may be a dense or sparse matrix or a linear operator for which a procedure is available to compute products *A***v** or *A^T^***w** on request for given vectors **v** and **w**. The gradient and Hessian of the convex function φ(**x**) are provided by another procedure for any vector *x* satisfying the bounds ***l*** ≤ ***x*** ≤ **u**. Greater efficiency is achieved if the Hessian is diagonal [i.e., φ(**x**) is separable].

Typically, 25–50 PDCO iterations are required, each generating search directions Δ**x** and Δ**y** for the primal variables **x** and the dual variables **y** associated with *A***x** + *D*_2_**r** = **b**. The main work per iteration lies in solving a positive-definite system


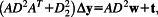
(11)

and then Δ**x** = *D*^2^(*A^T^*Δ**y** − **w**), where *D* is diagonal if φ(**x**) is separable. As **x** and **y** converge, *D* becomes increasingly ill conditioned. When *A* is an operator, an iterative (conjugate-gradient type) solver is applied to Eq. [11], and the total time depends greatly on the increasing number of iterations required by that solver (and the cost of a product *A***v** and a product *A^T^***w** at each iteration).

To solve problem [9] with a general dictionary *B*, we would work with **c** = **c**_1_ − **c**_2_ (where **c**_1_, **c**_2_ ≥ 0) and apply PDCO to Eq. [10] with the following input and output:


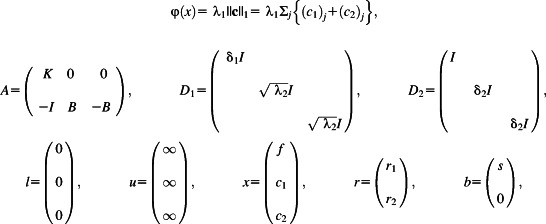


where δ_1_ and δ_2_ are small positive scalars (typically 10^−3^ or 10^−4^), ***r***_1_ represents ***r*** in Eq. [9], and ***r***_2_ will be of order δ_2_. For certain dictionaries, we might constrain ***c*** ≥ 0, in which case, ***c*** = ***c***_1_ above and ***c***_2_ = 0.

### LR-NMR Measurements

LR-NMR experiments were performed on a Maran Ultra bench-top pulsed NMR analyzer (Oxford Instruments, Witney, UK), equipped with a permanent magnet and an 18-mm probe head, operating at 23.4 MHz. Samples were measured four times to test the repeatability of the analysis. Prior to measurement, samples were heated to 40°C for 1 h and then allowed to equilibrate inside the instrument for 5 min. In between measurements, the instrument was allowed to stabilize for an additional 5 min.

The CPMG sequence was used with a 90° pulse with 4.9 ms, echo time (τ) of 100 ms, recycle delay of 2 s, and 4, 16, 32, or 64 scans. For each sample, 16,384 echoes were acquired. Following data acquisition, the signal was phase rotated and only the main channel was used for the analyses.

### RI-WinDXP

Distributed exponential fitting of simulations and real LR-NMR data were performed with the WinDXP ILT toolbox 21. Data were logarithmically pruned to 256 points prior to analysis, the weight was determined using the noise estimation algorithm, and logarithmically spaced constants were used in the solution.

### SNR Calculations

SNR consisted of taking the ratio of the calculated signal and noise. The signal was calculated as the maximum of a moving average of eight points. For the noise calculation, the last 1,024 echoes were chosen and corrected using the slope and intercept of the noise (χ*_i_*) versus the number of echoes, and the noise was calculated from the following equation:


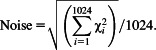
(12)

### Stability

Signal stability was determined using the coefficient of variation (cv) calculated as follows:


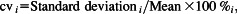
(13)

where the mean and standard deviation were calculated from four repeated measurements, and *i* = 1:256 is the distribution value. To get a measurement of the signal stability and disregard the noise, mean cv calculations were performed on the data that were higher than 25% and 10% of the maximum signal. Maximum cv values of around 15% were considered to give acceptable stability.

## V. RESULTS AND DISCUSSION

The new algorithm was extensively tested and calibrated using simulated data computed with an in-house Matlab function library. The objective of the simulations was to determine the accuracy and resolution of the analyzed spectra when compared with the noise-free simulated signal. In addition, simulations were used to determine universal, robust regularization coefficients that provide accurate and stable solutions for a broad range of signal types and SNR levels.

Two types of signals were simulated: i) a broad-peak signal and ii) a signal with narrow peaks that progressively become closer. The broad-peak signal was chosen as a typical *L*_2_ solution of an oil sample, with varying noise levels. The narrow-peak signal consisted of three peaks, artificially constructed according to a Gaussian distribution, with varying widths, signal strengths, and noise levels. Five types of narrow signals were used with the intrinsic *T*_2_ values described in Table 1.

**Table 1:** Intrinsic *T*_2_ Values of the Simulated Narrow-Peak Signal (ms)

	Intrinsic *T*_2_ of Peak 1	Intrinsic *T*_2_ of Peak 2	Intrinsic *T*_2_ of Peak 3
Signal 1	1.44	21.54	323.45
Signal 2	2.25	21.54	205.93
Signal 3	3.54	21.54	131.11
Signal 4	5.56	21.54	83.48
Signal 5	8.73	21.54	53.15

An additional narrow two-peak simulation, with peaks of varying widths that progressively become closer, was used to evaluate the resolution of the PDCO algorithm. In the simulations, a peak with an intrinsic *T*_2_ value of 81.54 ms was kept constant and another peak was gradually brought closer (27.53, 30.03, 32.75, 35.73, 38.97, 42.51, 46.36, 50.57, 55.16, 60.16, 65.62, and 71.58 ms). Similar peak widths were used in the two narrow-peak simulations, using four Gaussian functions with standard deviations 2, 3, 4, and 5.

### Calibration

As previously mentioned, it has been well established in the literature that the ILT is a notorious and common ill-conditioned inversion problem, whose direct inversion is unstable in the presence of noise or artifacts. Choosing an appropriate regularization method is therefore crucial for the establishment of an accurate and stable solution. In the experiments below, we used the simplest dictionary *B* = *I* and applied PDCO to the problem


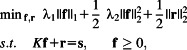
(14)

with λ_1_ = α_1_β/SNR and λ_2_ = α_2_/SNR, where *K_ij_* ≥ 0, max *K_ij_* = 1, and β = ||**s**||∞. Dividing the regularization coefficients by SNR provides calibration with respect to the signal strength, as less regularization is needed for larger SNR. Making λ_1_ proportional to β gives robustness with respect to scaling or normalization of the signals. These two kinds of robustness were tested and validated with a high level of certainty throughout the simulations, by ensuring that a single set of chosen values for α_1_ and α_2_ provides stable and high-quality solutions for different levels of noise or signal strengths. The method's robustness was validated for a minimum SNR value of 150. For much lower SNR values, larger α_2_ is recommended to prevent peak-splitting artifacts.

We believe that a tailored overcomplete dictionary *B* with a variety of peak widths and locations can significantly improve the results, as suggested by preliminary experiments. This remains for future study.

Calibration of α_1_ and α_2_ was performed using the simulated narrow-peak signals. For each simulated signal, a grid search was performed for the α_1_ and α_2_ values that gave the smallest error relative to the known solutions (min ||**f** − **x**||_2_, where **x** is the noise-free signal and ***f*** is the reconstructed signal). It was verified that the optimal results based on the residual *L*_2_ norm criteria were consistent with the decision of an expert using visual inspection.

**Figure 1:**
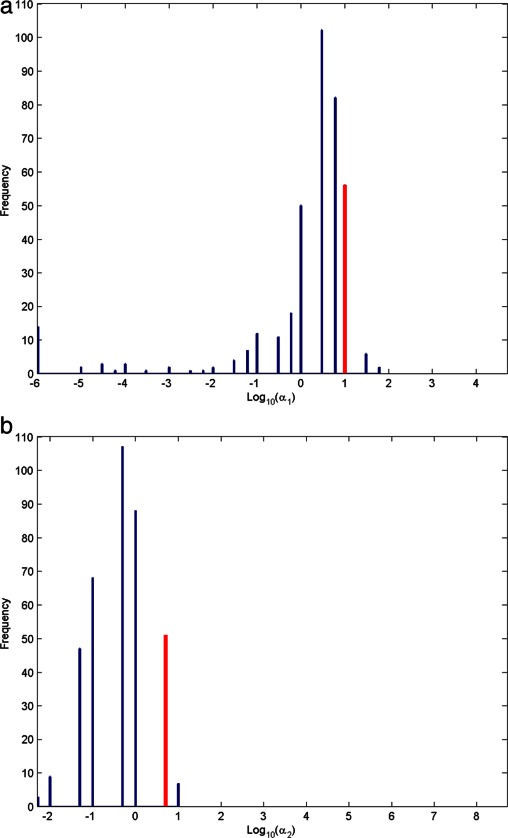
Histograms of the optimal (a) log_10_(α_1_) and (b) log_10_(α_2_) values that were obtained in the regularization parameters calibration simulation study. The universal log_10_(α_1_) and log_10_(α_2_) values chosen for the calibration are marked in red.

Figures 1(a,b) show histograms of the log_10_(α_1_) and log_10_(α_2_) values. As can be seen, optimal values of both αs were found in a relatively small range (the *x*-axis shows the entire range that was used for screening). Based on the histograms, the most common values chosen were α_1_ = 3 and α_2_ = 0.5, and this would be the natural choice for the calibration. The larger values (especially for α_2_) were mostly chosen for the widest peaks and low SNR values. Therefore, to establish a conservative calibration that also gives a stable solution for wide peaks and very low SNR values, 10 and 5 were ultimately chosen as the optimal α_1_ and α_2_ (marked in red on the histograms). As shown in the following examples, this choice of universal coefficients provides accurate and stable results for a wide range of signals and scenarios.

**Figure 2:**
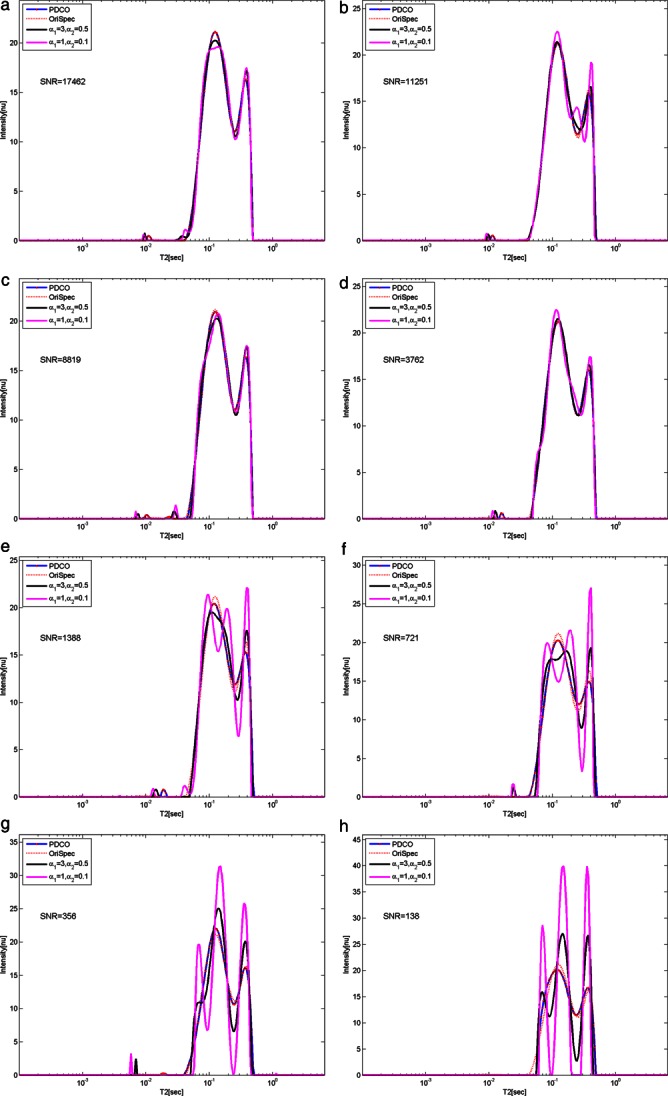
Comparison of representative PDCO analyses of the broad-peak signal simulations, using the universal regularization values for α_1_ and α_2_ (PDCO), the original simulated signal (OriSpec), and two less conservative choices for these parameters. The results are ordered by descending SNR values (a)–(h). The original noise-free simulated spectrum is shown for reference.

**Figure 3:**
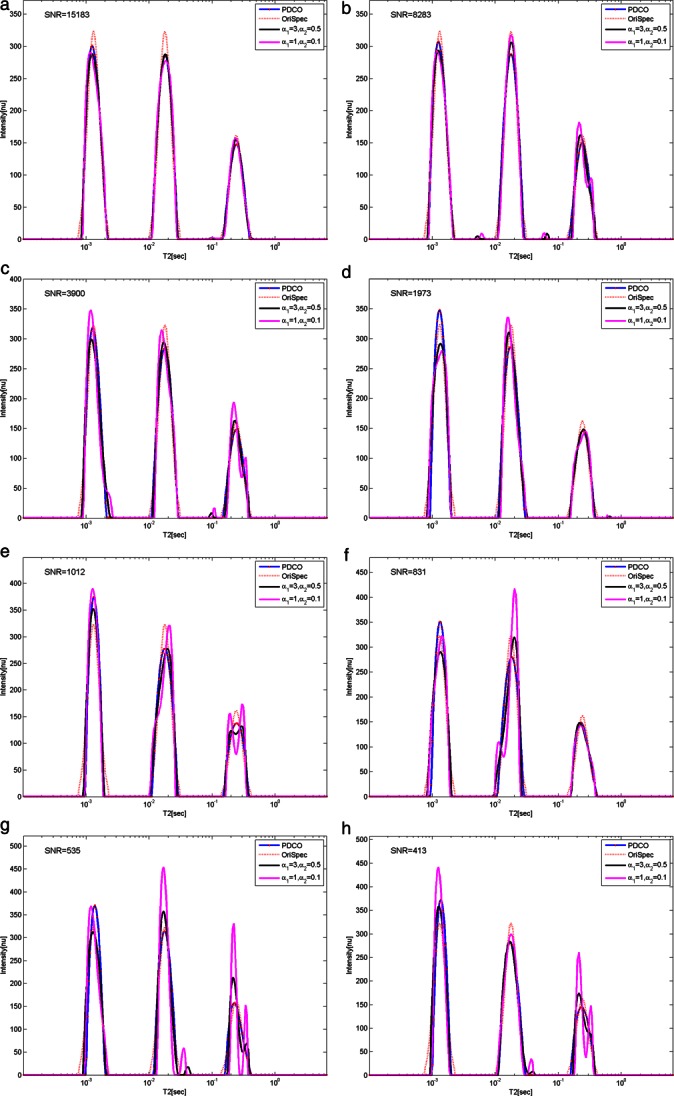
Comparison of representative PDCO analyses of Signal 1 simulations with wide peaks, using the universal regularization values for α_1_ and α_2_ (PDCO), the original simulated signal (OriSpec), and two less conservative choices for these parameters. The results are ordered by descending SNR values (a)–(h). The original noise-free spectrum is shown for reference.

The effect of under-regularizing the noisy signal was mostly stressed for the broad-peak simulations and the widest-well-separated peak simulations. These are more challenging factors to reconstruct an accurate and stable manner when compared with narrow peaks. Figures 2 and 3 compare representative PDCO analyses of the broad-peak simulations and Signal 1 simulations using wide peaks, respectively. The analyses were performed using the universal regularization values for α_1_ and α_2_ (PDCO) and two less conservative choices for these parameters. As shown, the relatively conservative choice of the proposed universal regularization parameters provides very good reconstruction of the broad peaks, even for low SNR levels. It is also important to note that decreasing the regularization parameters below the recommended universal values, especially α_2_, leads to the formation of spurious peaks that result in very different and unstable solutions when the SNR value is low. On the other hand, when the SNR value is high (according to these results approximately above 1,000), a slight decrease of α_1_ and α_2_ values does not degrade the solution. This, however, can lead to a dramatic change in resolving narrow adjacent peaks, which are more affected by the broadening effect of choosing a conservative choice for λ_2_ (data not shown).

### Resolution Analysis

To determine the resolution limit of the proposed method, we applied the narrow two-peak signal simulation with peaks of varying widths that progressively become closer. This procedure was carried out for four different SNR levels. The *T*_22_/*T*_21_ results (ratio of intrinsic *T*_2_ values of the two peaks) are summarized in Table 2.

**Table 2:** Resolution Analysis of a Two-Peak Signal Simulation with Four Widths Depending on SNR

		Peak 1		Peak 2		Peak 3		Peak 4	
					
	α_2_	5	0.5	5	0.5	5	0.5	5	0.5
SNR									
10,000		1.61	1.48	1.61	1.61	1.76	1.76	1.92	1.92
1,000		1.92	1.61	1.92	1.61	1.92	1.76	1.92	1.92
100		2.28	1.76	2.28	1.76	2.28	1.76	2.49	1.92
50		2.71	1.76	2.28	1.92	2.28	2.28	2.28	1.92

For each peak width and SNR level, the resolution limit was determined by marking the smallest separation in between the peaks for which the PDCO algorithm succeeded in separating the two peaks (separation was determined based on identification of peaks maxima). This procedure was repeated twice, one time using the conservative universal values for the regularization coefficients and another time using a lower α_2_ value.

It is important to note that both the peak width and SNR value have a major effect on the determination of resolution. The wider the peaks or the lower the SNR, the higher is the ratio of *T*_22_/*T*_21_ values that can be resolved. It has been shown that a Tikhonov regularization algorithm for a double exponential with *T*_22_/*T*_21_ > 2 can be reliably resolved if SNR > 1,000 (*6*). These results are in excellent agreement with those achieved using PDCO and the universal set of α_1_ and α_2_ values. As expected, with the less conservative regularization, the resolution limits were markedly improved, especially for narrow peaks.

### Comparison Between WinDXP and PDCO Results for Simulated Data

Distributed exponential settings of simulations were performed with the WinDXP ILT toolbox 21. To compare PDCO with the WinDXP solutions on the same simulated data, an in-house Matlab script was used to transform the simulated signals into the proper file format to be read by the WinDXP program. In addition, to remove uncertainties in the choice of regularization of WinDXP is unknown, and to demonstrate the influence of the *L*_1_ regularization component, relaxation time distributions were compared for PDCO with α_1_ = 0 and the universal value for α_2_, as determined by calibration.

**Figure 4:**
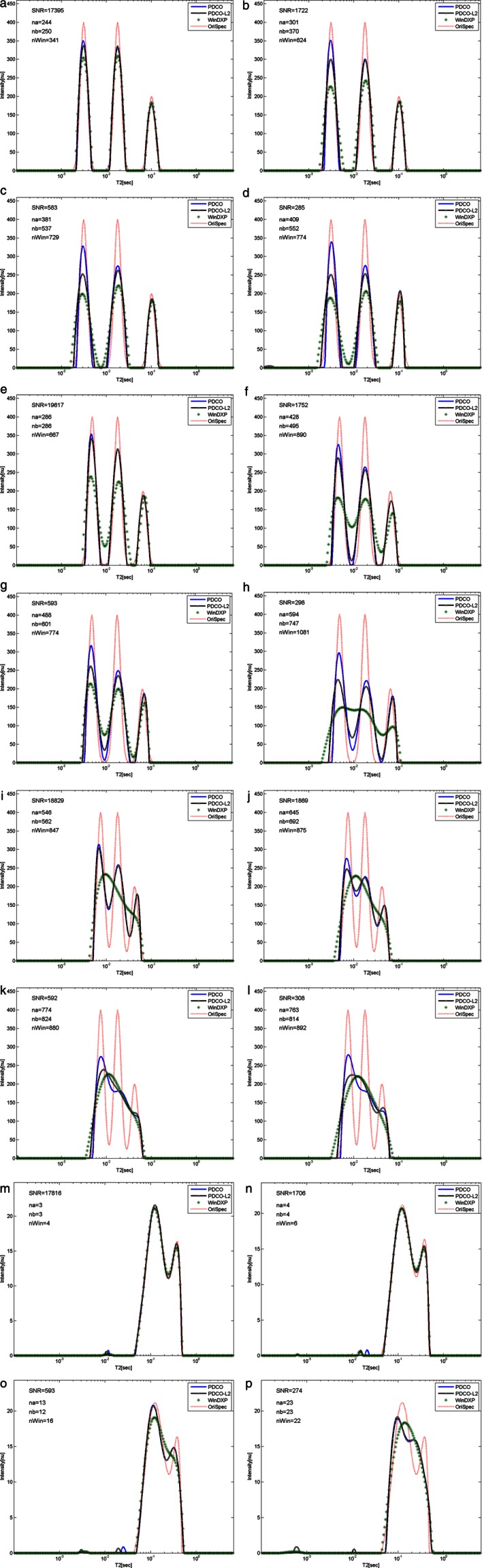
A: Comparison of representative simulation analyses using the PDCO universal regularization values for α_1_ and α_2_ (PDCO), PDCO with α_1_ = 0 and universal α_2_ (PDCO-L2), and the WinDXP (WinDXP) solutions. Relaxation time distributions for the narrow-peak simulations Signal 3 and Signal 4 are ordered by descending SNR values (a)–(d) and (e)–(h), respectively. The original noise-free simulated spectrum is shown for reference. na, nb, and nWin are the norm of the error relative to the known solutions for the PDCO, PDCO-L2, and WinDXP analyses, respectively. B: Comparison of representative simulation analyses using the PDCO universal regularization values for α_1_ and α_2_ (PDCO), PDCO with α_1_ = 0 and universal α_2_ (PDCO-L2), and the WinDXP (WinDXP) solutions. Relaxation time distributions for the narrow-peak simulations Signal 5 and the broad-peak signal are ordered by descending SNR values (i)–(l) and (m)–(p), respectively. The original noise-free simulated spectrum is shown for reference. na, nb, and nWin are the norm of the error relative to the known solutions for the PDCO, PDCO-L2, and WinDXP analyses, respectively.

**Figure 5:**
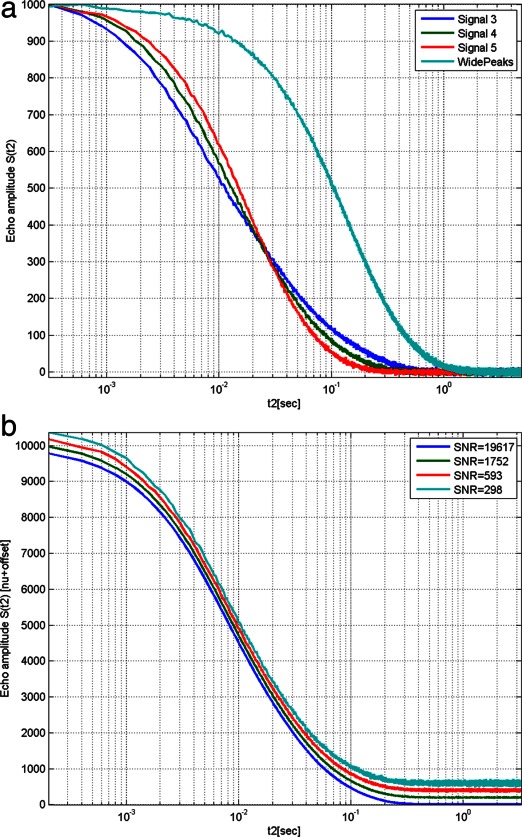
Logarithmic plots of the combined time-domain signals shown in Fig. 4 for (a) an SNR value of ∼300 (the echo amplitude of each signal was normalized to its highest value for simplicity of comparison) and (b) four different SNR values for Signal 4 (an offset was added to each relaxation curve for simplicity of comparison).

Figures 4(a–p) compare representative simulation analyses using the PDCO-established universal regularization values for α_1_ and α_2_ (PDCO), PDCO with α_1_ = 0 and universal α_2_ (PDCO-L2), and the WinDXP (WinDXP) solutions for four types of simulations and SNR values. Combined logarithmic plots of representative time-domain signals at different SNR values used for the comparison are shown in Figs. 5(a,b).

In the case of the wide-peak simulations, WinDXP and PDCO both solve relatively well [Figs. 4(m–p)]. The superiority of the PDCO solution over WinDXP is clearly demonstrated, especially for close and noisy signals. Based on the intrinsic *T*_2_ values of the narrow simulated peaks, a noted decrease in resolution is to be expected only for SNR < 1,000 of the closest peaks simulation (*T*_22_/*T*_21_ = *T*_23_/*T*_22_ = 2.47). This is in excellent agreement with the resolution analysis. Interestingly, even for a high SNR signal of 18,829, WinDXP cannot resolve any of the close peaks, and instead results in a very wide distribution [Fig. 4(i)].

In addition, with the *L*_1_ regularization term eliminated by setting λ_1_ = 0, the PDCO results are significantly better than for WinDXP. This may be due to a more conservative choice of calibration of the WinDXP, but perhaps more to the superior accuracy and numerical stability of the PDCO solver. In the latter case, PDCO would be a preferred solver even for traditional *L*_2_ regularization. It is also evident that the *L*_1_ regularization term improves the quality of the reconstruction results especially for low SNR. On the other hand, it has no apparent additional contribution to the solution of broad-peak signal simulations beyond the *L*_2_ term [Figs. 4(m–p)].

Despite introduction of the *L*_1_ term, the conservative calibration of PDCO leads to peak broadening, even at high SNR values [e.g., Fig. 4(i)]. This can lead to inaccurate conclusions regarding the physical and/or chemical microstructure organization. Based on extensive simulations of different types of signals and noise realizations, we feel confident in suggesting that the calibration can be moderated when SNR is high to search for a more general truth. This is shown in the next section using an oil sample whose true distribution is unknown.

### Comparison and Repeatability Analysis of WinDXP and PDCO Relaxation Time Distributions of a Real Rapeseed Oil Sample

**Figure 6:**
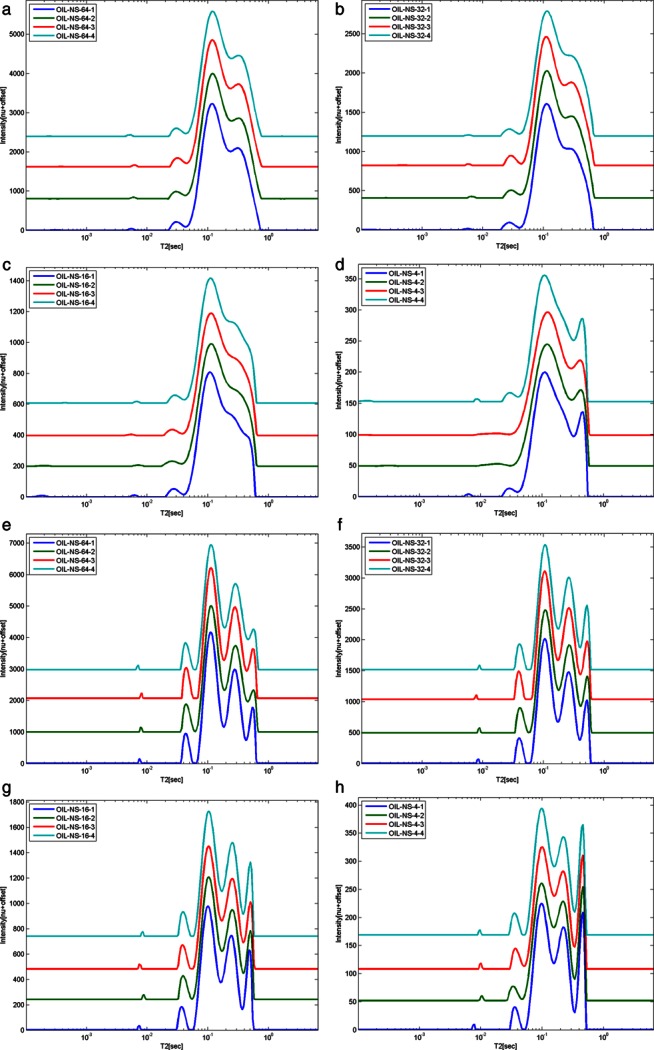
Comparison of WinDXP (a)–(d) and PDCO using the universal regularization values for α_1_ and α_2_ (e)–(h) solutions on a real LR-NMR dataset acquired from an oil sample. The results are ordered by descending number of scans (descending SNR).

**Figure 7:**
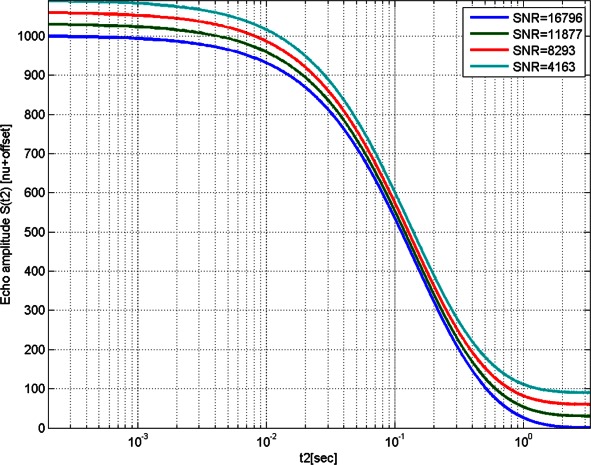
Logarithmic plot of the combined time-domain signals of a rapeseed oil sample acquired using 64, 32, 16, and 4 scans. For each NS value, only one representative signal and its SNR value are shown. The echo amplitude of each signal was normalized to its highest value, and an offset was added to each relaxation curve for simplicity of comparison.

Preliminary analysis of biological relaxation data acquired using LR-NMR is presented in Figs. 6(a–d and e–h) for WinDXP and PDCO, respectively. Here, a rapeseed oil sample was chosen as the model for comparison. The solutions are ordered by descending SNR values, based on the number of scans acquired (NS = 64, 32, 16, and 4, respectively). To test repeatability of results, measurements were separately acquired four times for each NS value. Combined logarithmic plots of representative time-domain signals at different SNR values used for the comparison are shown in Fig. 7.

Two other authors have presented broad-peak distributions, like the ones presented here for the WinDXP solutions [Figs. 6(a–d)] on pure avocado 22 and palm 23 oil samples. These were also analyzed using WinDXP. In contrast, the PDCO solutions have four distinct, moderately resolved peaks. As for this data, the original solution is unknown, these results raise the question of improved resolution versus the risk of introducing false peaks. To increase confidence in these results, we would like to point out several facts:

1. As previously shown, with the universal regularization values for α_1_ and α_2_ in PDCO on different types of simulations, no spurious peaks were introduced into the solution. More precisely, in Figs. 2(a–d), we presented the PDCO solutions of a broad-peak signal simulation whose SNR values closely meet those presented here. Based on these results, provided the broad-peak signal is the true signal, no peak splitting is to be expected in the solution.
2. In the case of under-regularizing, unstable solutions are to be expected in the form of spurious peaks that are due to random noise (as shown in Figs. 2 and 3). As can be seen, all four repetitions of the PDCO solutions, per and between NS values, are highly repeatable and stable.
3. From a physical point of view and in accordance with the resolution analysis, the minimum separation between peaks in the oil sample can in theory be accurately resolved for SNR > 1,000 (intrinsic *T*_2_ values at 46, 114, 277, and 542 ms).

**Figure 8:**
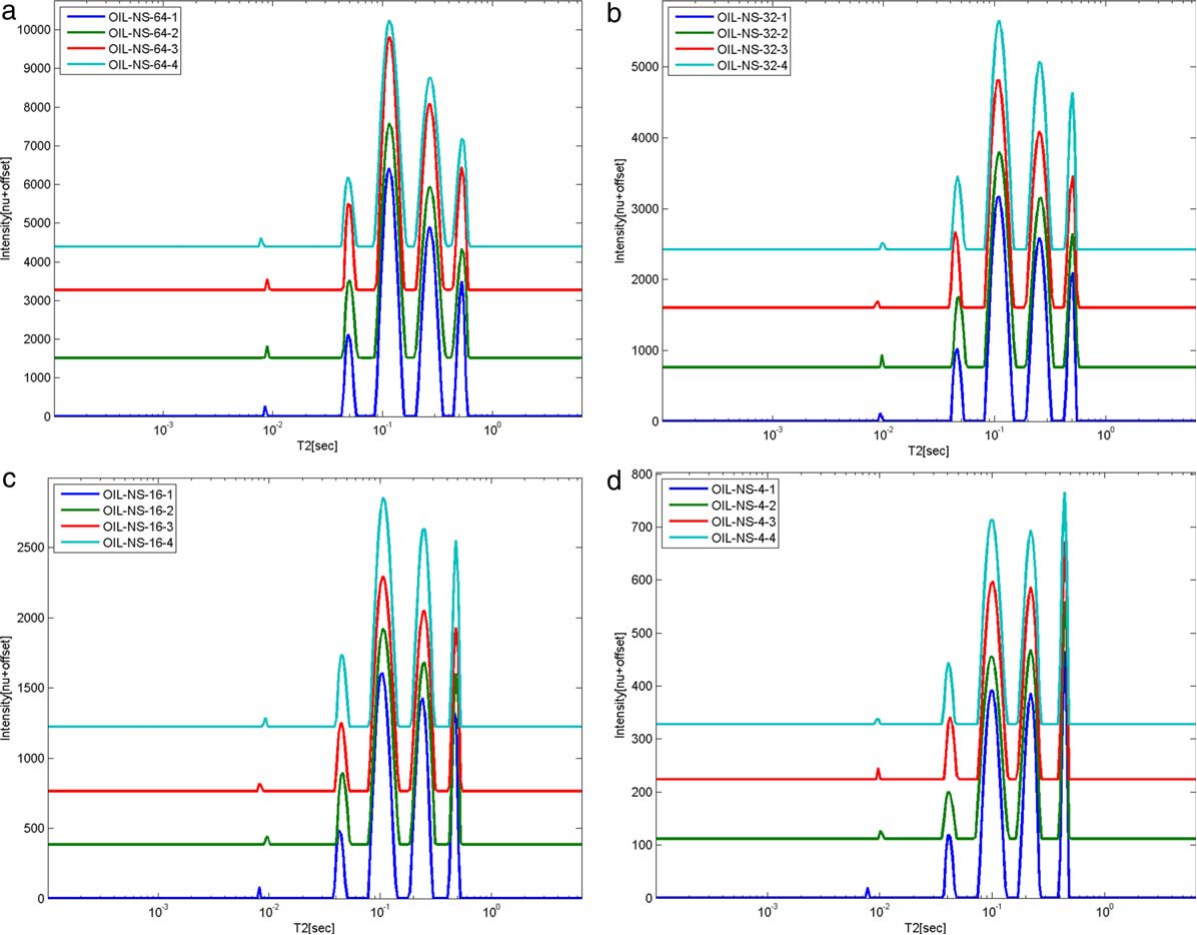
Relaxation time distributions analyzed using less conservative PDCO (α_2_ = 0.5) on the same real LR-NMR dataset acquired from a rapeseed oil sample. The results are ordered (a)–(d) by descending number of scans (descending SNR).

Based on these arguments, it is our belief that the PDCO formulation provides better resolved relaxation time distributions and more accurate solutions. Moreover, as it was shown that the universal calibration values of α_1_ and α_2_ introduce a broadening effect to originally narrow peaks, we wanted to explore the effect of using a more moderate value for α_2_ (0.5) on the solution [Figs. 8(a–d) for decreasing SNR values]. We feel confident that the relatively high SNR values of the oil samples still allow us to remain in the safe zone in light of the risk of under-regularizing. As shown, the results are highly repeatable, even for the different SNR values, and look very similar to those analyzed using the more conservative α_2_ value, in that no splitting or spurious peaks appear in the distribution.

Assuming that the PDCO solution for the rapeseed oil sample is more accurate than the accepted WinDXP one, an explanation for the different peaks is still needed. Their assignment is not an obvious task, as several authors struggled with this question even for the bimodal distribution, and did not provide a conclusive answer. Marigheto et al. 24 speculated that it arises from molecules of differing mobility, such as the oleic and palmitic constituents, or from nonequivalent proton pools of different mobility, such as those on methyl and olefinic groups. Adam-Berret et al. 25 suggested that this may be due to inhomogenous relaxation rates for the protons along the side chains or inhomogenous organization of the triacylglycerols in the liquid with intermolecular interactions. We intend to address this question and already initiated a thorough research plan in this area using the new PDCO algorithm. However, it is not in the scope of the current work and will be explored in a separate publication.

### Stability Analysis

A stability comparison of the relaxation time distributions calculated by the PDCO algorithm according to the coefficients determined by calibration (α_1_ = 10; α_2_ = 5), less conservative PDCO algorithm (α_2_ = 0.5), and WinDXP on the oil sample is shown. Data were analyzed using the four repetitions acquired using 4, 16, 32, and 64 scans. The comparison of the numerical stability of the two algorithms based on these results is shown in Table 3.

**Table 3:** Comparison of the Stability of WinDXP and PDCO Using the Universal Regularization Values for α_1_ and α_2_ and PDCO with a Less Conservative Choice of α_2_ Based on the Data Acquired on an Oil Sample

	Mean Coefficient of Variation That Exceeded the 25% Threshold			Mean Coefficient of Variation That Exceeded the 10% Threshold		
		
	WinDXP	PDCO	PDCO	WinDXP	PDCO	PDCO
	α_2_	5	0.5		5	0.5
NS						
4	3.8	3.6	5.0	7.2	6.7	10.9
16	2.4	6.6	13.3	2.9	8.0	20.0
32	1.7	4.1	7.5	2.2	5.6	13.7
64	1.4	5.7	5.8	1.7	7.2	10.5

The tabulated numbers are means of the cv*_i_* that exceeded the 25% and 10% thresholds.

As can be seen, both algorithms are highly stable for all cases except one where the mean cv exceeds the 15% maximum threshold determined for acceptable stability. Furthermore, mean cv lower than 10% was found for the two cutoff values and all NS for PDCO with universal α_1_ and α_2_. It is also shown that WinDXP has a slight advantage. This is probably due to the broad smooth distributions of the WinDXP when compared with the more resolved distributions of the PDCO, where small changes in the solutions are notably more pronounced in the cv parameter. As WinDXP smoothes the solution, it is substantially less prone to random changes that arise from noise in repetitions of the same signal. This same smoothing, however, leads to a large bias in the solution, as shown before for the reconstructed simulated signals.

It is worth noting that variability in the solutions may originate from instabilities in the acquired signals. From a preliminary experiment of signal acquisition using LR-NMR, we found that the PDCO algorithm is more sensitive to measurement imperfections than WinDXP. We concluded that in order to achieve high-quality repeatable results using PDCO, the offset frequency (O1) should be calibrated prior to each measurement, and the instrument should be allowed to stabilize between data acquisitions.

## VI. CONCLUSIONS

Effective solution of the inverse LR-NMR problem requires an integrated multidisciplinary methodology. Our proposed integrated approach, including validation of analyses by simulations, testing repeatability of experiments, and validation of the model and its statistical assumptions, has led to the development of an improved tool for analyzing LR-NMR relaxometry data. Improvement was achieved by 1) introducing an *L*_1_ regularization term to the mathematical formulation, 2) adjusting and applying the accurate and numerically stable PDCO solver, and 3) choosing universal coefficients for the calibration based on extensive simulations with different types of signal and SNR values. We believe that this new methodology could be applied to the two-dimensional cross-correlation problem 26,27 to improve the peaks distortion problem mentioned by Venturi et al. 28.

## BIOGRAPHIES

**Paula Berman** obtained her B.Sc. in Biotechnology Engineering in 2008 at Ben Gurion University of the Negev, Beer Sheva, Israel, and is currently a doctoral student at the Department of Environmental Engineering at the same institute. Paula's research is conducted under the supervision of Prof. Zeev Wiesman and involves development of new low-resolution NMR tools and their application to the field of biodiesel.

**Ofer Levi** received his PhD in Scientific Computing and Computational Mathematics from the Stanford University in 2004. Levi has been a lecturer in the Department of Industrial Engineering Management of Ben-Gurion University since 2004. Levi's main interests include mathematical and statistical modeling of physical systems, inverse problems, compressed sensing, signal and image processing, parallel and distributed computing, biologically inspired analysis methods, and physiological signal processing.

**Yisrael Parmet** is a senior lecturer at the Department of Industrial Engineering and Management of Ben-Gurion University. He holds a B.A. in Economics and Statistics and M.Sc. and Ph.D. degrees in statistics from the Tel-Aviv University. He specializes in area design of experiments and statistical modeling. During his studies for a master and doctorate degrees, he served as research assistant at the statistical laboratory at the Department of Statistics and OR, Tel Aviv University, which granted him knowledgeableness in practical data analysis. In 2007–2008, Parmet was a visiting professor at Department of Dermatology and Cutaneous Surgery in the UM Miller School of Medicine.

**Michael Saunders** specializes in numerical optimization and matrix computation. He is known worldwide for his contributions to mathematical software, including the large-scale optimization packages MINOS, SNOPT, SQOPT, PDCO, and the sparse linear equation solvers SYMMLQ, MINRES, MINRES-QLP, LUSOL, LSQR, and LSMR. He is a research professor at Stanford University in the Department of Management Science and Engineering (MS&E) and a core member of the Systems Optimization Laboratory (SOL) and the Institute for Computational and Mathematical Engineering (ICME). In 2007, he was elected Honorary Fellow of the Royal Society of New Zealand, and in 2012, he was inducted into the Stanford University Invention Hall of Fame.

**Zeev Wiesman** is an expert in plant lipid biotechnology. His research group studies are focused on advanced multispectral technologies development including low-resolution NMR and mass spectrometry for monitoring, evaluation, and prediction modeling of biomass feedstocks and processes most relevant for biofuels industry. In the past 20 years, he has published about 100 manuscripts, chapters in books, a book, and patents. He is heading an Interdisciplinary Energy Engineering program of research students at Faculty of Engineering Sciences at Ben Gurion University of the Negev, Beer Sheva, Israel.
